# Editorial: Beyond formal models of reasoning about explanations

**DOI:** 10.3389/fpsyg.2024.1358934

**Published:** 2024-01-30

**Authors:** Barbara Koslowski, Igor Douven, Amy Masnick, Karolina Krzyzanowska, Clark Chinn, Bradley Morris

**Affiliations:** ^1^Department of Human Development, Cornell University, Ithaca, NY, United States; ^2^French National Centre for Scientific Research (CNRS), Université de Paris, Paris, Ile-de-France, France; ^3^Department of Psychology, Hofstra University, Hempstead, NY, United States; ^4^Institute for Logic, Language, and Computation, University of Amsterdam, Amsterdam, Netherlands; ^5^Graduate School of Education, Rutgers University-Newark, Newark, NJ, United States; ^6^Department of Learning Sciences and Educational Psychology, Kent State University, Kent, OH, United States

**Keywords:** abduction, explanation, scientific reasoning, cognition, formal models

Formal models of reasoning typically consist of strategies that are treated as though they were algorithms, that is, as though their application will yield a conclusion that pretty much guarantees an accurate outcome. In the psychological literature, a common example consists of identifying the cause of an event by identifying which events covary with it and that are also temporally prior to and physically contiguous with it. This strategy acknowledges that, in some cases, the results are probabilistic rather than definitive, but it ignores an important limitation that is captured by the admonition that correlation does not guarantee causation.

Of course, as the articles in this issue recognize, one of the limitations is that nearly all reasoning is fairly obviously influenced by “background information.” And crucially, background information can include theory or explanation.

Philosophers and psychologists talk about paradigmatic examples of the importance of explanation in science by using the description “Inference to the Best Explanation” (IBE) often referred to as “abduction,” a framework based on actual scientific practice rather than formal models—and a framework in which (as it does in science) explanation plays a crucial role (Harman, 1965; Lipton, 1991, 1993; Douven, 1999, 2011; Capaldi and Proctor, 2008; Koslowski, 2012a,b, 2017). The articles in this issue examine the ways in which explanation functions in more contexts than just scientific reasoning.

For example, Vasil and Lombrozo find that explanations that posit a mechanism vs. those that merely mention a covariation are differentially affected by subsequent information about the strength of the covariation and about whether the mechanism is full, that is, detailed. To be sure, identifying a covariation can be one type of explanation; noting that smoking covaries with cancer, for example, is one sort of explanation for why cancer occurs. But Vasil and Lombrozo find, in addition, that the two types of explanations function differently; although both types facilitate narrow generalization, mechanism information facilitates broad generalization.

Three of the contributions extend the notion of an explanation to include a broad, cultural framework. Niiya et al. note the role of explanation in accounting for cross-cultural differences. For example, an account based only on covariation might note that Japanese are less likely than Americans to offer help to a stranger. However, Niiya et al. provide an explanation for that cultural difference, namely, that Japanese are the more likely to consider, not what a *helper* would want in that situation (which is what many Americans would do), but rather what the stranger being helped would want. For example, the stranger might not want help that could potentially lead to embarrassment. Thus, the presence of an explanation provides a more detailed account of this particular cultural difference.

Wang et al. focus on the cultural differences of collectivism vs. individualism involved in pandemic control and find that the difference is mediated by actual policy (along with several other factors such as education, age, etc.). The finding that policy mediates (or in the present terms is the mechanism or explanation that accounts for the collectivism vs. individualism difference) fleshes out the initial account based on simple covariation.

Tirasawasdichai et al. find that cultural learning can result from simply watching foreign movies and TV series, which in turn leads to intercultural engagement, including cultural acceptance of the target culture.

The framework provided by sexism also constitutes an explanation in the extended sense. Konig and Heine find that people can learn to detect sexism after a brief intervention, but that people also (problematically) then “detect” sexism when it is not present.

Labotka and Gelman find that different explanations (in this case, folk theories and scientific theories of disease transmission) can and do often co-exist. In addition, people often invoke multiple explanatory frameworks (biological, mechanical, psychological) for a single phenomenon. Furthermore, and especially relevant to the current issue, background information can play a role in which explanation takes center stage in a situation.

Finally, Fedyk et al. offer a provocative critique of one of the limitations of IBE. They argue that their alternative, “inference to the best action” (IBA) provides a more accurate account than does IBE of actual clinical expertise and practice, because, in addition to the background information known by different clinicians, the “background *environment*” — the physical space in which reasoning occurs — plays a role in establishing the reliability of extremely complex patterns of reasoning. For example, the strategy of using chest compressions in an emergency room does not rely on invoking theory during the procedure, but rather on a process that bundles together a dynamic repertoire of actions that have been successful in the past, such as the ability of a team to fluidly rotate among members who have become exhausted from providing the compressions and administering the appropriate medications at the correct time. If the Fedyk et al. analysis is correct and holds true of other kinds of expertise, then IBA needs to be considered alongside IBE in debates about the cognitive foundations of abduction.

In summary, the articles in this issue identify some of the important ways in which explanation does and does not play a central role in reasoning in a variety of contexts. The centrality of explanation is not restricted to science.

## Author contributions

BK: Writing – review & editing. ID: Writing – review & editing. AM: Writing – review & editing. KK: Writing – review & editing. CC: Writing – review & editing. BM: Writing – review & editing.
